# The Use and Abuse of Pessaries

**Published:** 1885-12

**Authors:** Virgil O. Hardon

**Affiliations:** Atlanta, Ga.


VOL. II. DECEMBER, 1885. NO. 10.

### Original Communications.

### THE USE AND ABUSE OF PESSARIES.

BY VIRGIL O. HARDON, M. D., ATLANTA, GA.

READ BEFORE THE ATLANTA SOCIETY OF MEDICINE, OCTOBER 20, 1885.

It is a trite observation that there exists a strong tendency in medical opinion to run to extremes of approval and condemnation of modes of treatment whose value has not been definitely determined. The eagerness with which a new discovery is hailed, and which is based upon the success attained by the originator operating upon selected cases with the skill and experience incident to a thorough study of the subject, in time gives way to an opposition, based upon disappointment and failure in the hands of novices, who apply the newly-discovered method without discrimination or skill. In no department of medicine are these facts more frequently and forcibly exemplified than in that of diseases of women. During the establishment of a scientific system of gynecology within the last half century, nearly all the great operations which mark the rapid strides of that branch of study have run the gauntlet of—first, undue praise; then, adverse criticism; and finally, an intelligent and discriminating adoption.

It is not surprising, therefore, that great diversity of opinion should be found among eminent authorities in regard to the proper application of mechanical treatment for displacement of the uterus, or that, after human ingenuity has been apparently taxed to its utmost limit in devising new pessaries, as diverse in their shapes as in their degrees of usefulness, a tendency should be manifested in modern gynecology to abandon the use of the pessary altogether, or to return to its primitive form, which consists of a medicated tampon of cotton, or some similar soft material. This tendency can be seen running through all the current literature of the subject, although the great leaders in gynecology will be found to occupy the middle ground of conservatism. Thomas, for instance, in speaking of pessaries, says: "Were I asked at the present moment whether I believed that, in the aggregate, they accomplished more good or evil, I should be forced to give a doubtful reply."* Such is the general drift of opinion among authorities whose skill and experience commend their views to the most profound respect. A quarter of a century ago there was no such uncertainty of opinion, and its existence at the present day shows that a reaction has taken place, and a gradual approach to the opposite extreme may naturally be looked for.

But, in addition to this natural tendency toward a reaction, there is also another reason for the modification of the former belief in the efficacy of pessaries. With the increase of exactness in the diagnosis of uterine affections, and with the better adaptation of means to the cure of morbid conditions, there has grown up a closer observation of the results of treatment, and hence a disposition to scan more rigidly the efficacy of methods which tradition and custom have rendered sacred. The use of pessaries dates from the days of Hippocrates, and their value has, in former times, been looked upon as established beyond a question. They have been inserted in a perfunctory and routine way wherever there has been found a uterine displacement, and it can

*Diseases of Women. New York, 1880, p 67.

readily be conceived that, in unskillful hands, they have accomplished an immense amount of harm, which has never been placed to their credit.

Yet it was not until the inauguration of the improved methods, now employed in the investigation of uterine diseases, that the profession began to realize that they had been playing with a weapon as potent for evil as for good, and that the bad effects of an ill-fitting pessary had been often mistaken for symptoms of the disease under treatment. How many a pelvic cellulitis has been set up, how many an existing inflammation has been fatally aggravated by this means, can never be estimated. But that their name is legion may be inferred from the fact that such results are not unknown even in the most skillful hands at the present day. Meigs said, "Pessaries are necessary evils,"* and the forty years which have elapsed since he made this statement have only served to confirm its truth. But the evil is not necessarily inherent in the instrument itself, but lies oftenest in its misapplication. Emmet says, "I have never known a practitioner, who was able to fit a pessary properly, who was not also fully satisfied with the amount of benefit derived from its use."† This statement expresses only a part of the truth. It may be rendered complete by making the ability to recognize the indications for the use of a pessary an additional requisite to its satisfactory employment. My observation has convinced me that the number of cases in which a pessary is applied unnecessarily is fully as great as that in which it is applied unskillfully. In either case, the treatment must inevitably result in failure, and the physician condemns the instrument instead of its unwise application. From oft-repeated experiences of this kind, the opinion is gaining ground that the less the profession have to do with pessaries, the better will it be for themselves and for their patients. This opinion is, to a certain extent, a judicious one, for the proper application of a pessary requires an amount of skill and experience which is not possessed by the majority of general practitioners. Hence, the discouragement of their use would doubtless result in actual benefit. But it

*Females and their Diseases. Philadelphia, 1848, p. 144.
†Principles and Practice of Gynecology. New York, 1884, p. 302.

is manifestly illogical to condemn these instruments on account of their failure to accomplish good results in incompetent hands. Yet it appears that this is usually the chief ground on which their condemnation is based.

In order to apply a pessary properly, and thereby obtain grounds for an intelligent opinion as to its usefulness, certain requisites are indispensable, and their absence is the cause of many of the failures to achieve satisfactory results. They are—

1. A familiarity with the anatomy of the organs of the female pelvis.

2. A certain amount of mechanical skill.

3. A sufficient supply of pessaries from which to select.

4. A precise idea of what is to be accomplished by the pessary selected.

It is only within a few years that the anatomy of the female pelvis has been understood. One who looks in the older works on anatomy, obstetrics or gynecology, at the figures which are designed to represent the normal appearance of the pelvic viscera, and compares them with the most recent authorities on the subject, will be struck by the wide differences which present themselves. In the former, the vagina is represented as a dilated cylinder with a continuous curve from the vulva to the fundus uteri, with the concavity looking forward. The uterine, cervical and vaginal canals thus form nearly a half circle with no angles. The uterus has apparently no support from the surrounding parts, the perineal body is an extremely insignificant feature, and the vaginal promontory has no existence. On turning to the true anatomy of the pelvis, as demonstrated by recent investigators, there will be found an entirely different aspect of affairs. The utero-vaginal canal is a closed passage, its walls being everywhere in contact. It has two curves, the principal one, from the vulva to the os uteri, with its concavity looking backward, and the other at the cervical junction, forming a sharp angle forward of fully forty-five degrees. The perineal body is a large triangle occupying the greater part of the recto-vaginal space and forming, by its anterior convexity, the vaginal promontory, which plays an important part in the support of the womb. If a comparison be made between these two pictures, the former of which was regarded as correct until a very recent period, can it be wondered at that failure has so often attended the use of an instrument whose success is dependent entirely upon its mechanical adaptation to the vaginal canal? To a similar lack of anatomical knowledge may be attributed a considerable portion of the dissatisfaction attending the use of pessaries at the present day.

Without a certain amount of mechanical skill, it is impossible for any man to succeed in the treatment of uterine displacements by the use of pessaries. The problem presented is wholly a mechanical one, and the ingenuity of the gynecologist finds in this field ample scope for its fullest exercise. The problem of maintaining the position of the uterus by a support which shall take its bearings from the vaginal walls without undue pressure at any point is by no means a simple one, and it is only by the study of the mechanical conditions of each individual case that its solution can be successfully attempted. Emmet says he never uses two pessaries of exactly the same form and size, because he adapts each instrument to the individual case, as a tailor would a garment. "Fortunately," he adds, "there are many women who are able to tolerate an ill-fitting instrument without receiving injury, but they are not benefited, except it be by sheer good luck."* The fact that the pessary must conform to the vagina, and not the vagina to the pessary, is one which is often overlooked. Yet upon the recognition of this fact hangs success or failure. Hence, the physician must be able to appreciate all the mechanical conditions in the case before him, and to select or shape a pessary which will exactly meet those conditions, if he would obtain good results from its use.

The necessity of adapting the pessary to the individual case involves also the necessity of a large supply of pessaries from which to select. No one style of pessary is adapted to all cases of displacement, and the wider the range of the physician's resources in this direction, the more probable will it be that he will select the appropriate instrument for any particular case. He may very properly prefer a certain style of pessary for a certain

*Opus cit., p. 303.

class of cases. But even then he must adapt it to the individual peculiarities of the case before him, for it will be found that there is as much difference in vaginæ as in noses in respect to shape and size. I have succeeded in obviating, to a great extent, the difficulty often experienced in obtaining a pessary of the desired shape and size by using Sims' block tin rings, which can be obtained in all sizes, and which can be readily bent to any shape by the fingers, and will retain that shape in the vagina. Thus it is possible to meet all the various shades of difference which may present themselves, and to fit the instrument with an accuracy limited only by the mechanical skill of the operator.

But more important than all else is it that a correct idea should be formed of what is to be accomplished by the use of a pessary. The indications in the treatment of a displacement of a womb are two-fold: First, to obviate the evil effects of the displacement; second, to remove its cause. It is a common error to suppose that displacements can be cured by the use of pessaries, and the disappointment arising from this error is no small factor in the production of the prevalent dissatisfaction with their use. A displacement is never a disease *per se*, but is simply one of several symptoms arising from a common cause. This cause varies greatly, and the treatment must of necessity be as various as the cause. It is often easy enough to restore a womb to its place, keep it there by a well-fitted pessary, and then say to the patient, "You are cured." But in that case, only a single symptom has been relieved, while the cause still persists, and as soon as the mechanical support of the womb is removed, it will return to its abnormal position. Hence, it is not for removing the cause that the pessary is indicated. It is in fulfilling the first indication named that its proper province is found, viz., to obviate the evil effects. The injurious results of a displacement are due to—first, traction upon the ligaments; second, interference with the circulation in the womb; third, pressure or traction upon the bladder. These symptoms are not necessary accompaniments of every displacement. It is no uncommon thing to find an anteflexion, for instance, persisting for a long time without producing either of these results. It is only when the womb, from some other cause, settles down below its normal position in the pelvis that the evil effects appear. Emmet says, "To whatever cause the increase in size and weight of the uterus may be due, the organ will settle into the pelvis just in proportion to the additional burden."* This settling down of the womb in the pelvis usually arises from the same cause that produces the displacement. A lacerated cervix, for instance, will interfere with the involution of the womb after labor, and the organ will remain of greater than normal weight. When the patient gets on her feet, it sinks down in the pelvis because the normal equilibrium between the natural supports and the weight to be supported is destroyed. The pathological condition of subinvolution deprives the uterine tissues of their natural tonicity, and this deprivation, in connection with the increased weight of its body, renders it possible for the fundus to sink backward or forward in the direction of least resistance. There follows, as a result, traction upon the broad ligaments, causing inflammation in the cellular tissue, derangement of circulation due to constriction of the blood vessels going to the womb, and vesical irritation from pressure or from traction upon the vesico-vaginal septum, and the neck of the bladder. In such a case, all the symptoms are due to a common cause—laceration of the cervix.

The effect of a pessary upon the cause of this train of symptoms would be *nil.* But if the object of its use be to relieve the disagreeable symptoms only, that object will probably be accomplished. In using the pessary, it is necessary that the fact should never be lost sight of that the symptoms whose relief is sought are due to the settling down of the womb into the pelvis and to that alone. This may be demonstrated by lifting the cervix upon the finger in the vagina while the patient stands. Unless the womb be bound down by adhesions, it will be found that there is a certain point of elevation at which the traction upon the ligaments, the pressure upon the bladder and the obstruction of the circulation are relieved, as shown by freedom from pain and discomfort. If the womb can be held at that point, the symptoms are relieved, and it is for this purpose alone that a pessary

*Opus cit., p. 348.

can be advantageously used. Whoever expects to accomplish more than this will be disappointed.

A not uncommon error consists in so applying the pessary as to raise the womb above its normal position in the pelvis, thereby producing essentially the same symptoms that result from a corresponding degree of depression. Traction on the broad ligaments and the neck of the bladder, with derangement of circulation, will result as readily from displacement upward as from displacement downward. Hence, in applying a pessary, it is necessary to first ascertain the point of elevation at which the patient is most free from pain and discomfort. The pessary should be so fitted as to hold the womb at that point. Fritsch says that the normal mobility of the womb is such that it may be pushed upward an inch and a half without discomfort.* But my experience has taught me that any attempt to raise the organ much above its normal position in the pelvis will produce pain and will defeat the objects for which a pessary is applied. The normal position of the uterus has been the subject of a great deal of discussion and has never been satisfactorily determined, and of necessity it never can be determined, for it varies greatly in different subjects, and even at different times in the same subject. But when the point has been found at which the uterus exerts the least interference with surrounding parts, as shown by the degree of pain and discomfort, it may be concluded that its normal position has been determined for that individual case.

The one great contra-indication to the use of a pessary is the existence of inflammation in any part of the pelvic cavity. The merest tyro in gynecology would hesitate before applying a pessary during an attack of acute pelvic cellulitis. But the fact is not sufficiently appreciated that it is equally improper to do so as long as there is any tenderness or hardness in any part of the pelvis. In its normal condition, all parts of the vagina are soft and yielding to the finger, and pressure at any point will be borne without pain. Any deviation from this condition is an evidence of a pathological change. In the majority of cases which present themselves for treatment, the roof of the pelvis will be found to

*Review in *American Journal of Obstetrics*, July, 1881, page 707.

be tender to the touch, and this tenderness will be especially marked along the tract of the broad ligaments. The amount of tenderness may vary in degree from a slight pain when firm pressure is made up to such an extreme of sensitiveness that the patient will scream when the finger is introduced into the vagina. In addition to this tenderness it will usually be found that the whole or a part of the space surrounding the cervix is filled with a hard, unyielding deposit, which has been aptly likened to a board in the sensation which it communicates to the finger. This deposit holds the cervix firmly fixed in one position, and in a greater or less degree deprives the womb of its normal mobility. Such a deposit cannot exist unless inflammation has been present. The combination of this board-like hardness, with a greater or less degree of tenderness, constitutes evidence of the existence of chronic pelvic cellulitis. Whether this condition is one of true inflammation of the cellular tissue, it would be out of place to discuss here. It is sufficient to know that the condition is easily recognizable clinically, and while it exists the use of the pessary is contra-indicated. The reasons for this contra-indication are, first, because as a rule the pressure of a pessary under such circumstances cannot be comfortably endured by the patient ; second, because the unnatural immobility of the womb prevents its restoration to the normal elevation, in which alone the pessary can be of benefit ; third, because experience has shown that the pressure of a pessary under such circumstances is almost certain to convert the chronic cellulitis into an acute attack. Before a pessary can be applied without danger, every vestige of tenderness and hardness must be removed. The persevering use of copious vaginal injections of hot water, painting the whole surface of the cervix and vagina every three or four days with compound tincture of iodine, and the insertion daily into the vagina of a tampon of cotton saturated with glycerine, are the means which will be found best adapted to the accomplishment of this result. In some cases it will be necessary to employ these measures longer than in others,but if the perseverance of physician and patient be equal to the emergency, success will ultimately crown their efforts.

When this point is reached, and not till then, may the question of inserting a pessary be safely considered.

There are certain precautions to be observed in the employment of pessaries, without which their use cannot be free from danger. A pessary should never be allowed to remain in the vagina after it becomes in the least degree painful. The pain in such cases is nature's danger signal, which cannot be safely disregarded. The patient must, in all cases, be instructed to have the pessary removed as soon as she feels any discomfort from it ; or better still, she should be taught to remove it herself, and thus prevent the dangerous delay which might be unavoidable if she were obliged to resort to the physician. I never allow a patient to leave my office with a pessary in her vagina without first having seen her remove it unaided. In no other way can I feel assured that she is safe in wearing it. My instructions always are, "As soon as you feel any pain, remove the pessary and send for me at once." When sent for under such circumstances, I direct the patient to go to bed, and to use copious vaginal injections of hot water every two hours until all pain and tenderness have disappeared. I am satisfied that by this prompt treatment I have prevented attacks of acute pelvic cellulitis.

It is the duty of the physician to see and examine with the speculum, at least once a month, every patient wearing a pessary. Undue pressure upon the vaginal walls has been known to proceed to the extent of producing severe ulceration without sufficient pain to attract the attention of the wearer, and pessaries have been found imbedded in the vaginal walls, which had been inserted a score or more of years before and entirely forgotten. Neither of these results would be possible if the pessary were removed at each recurrence of the menses and a vaginal examination made before it was re-introduced.

During the wearing of a pessary, a copious vaginal injection of hot water should be used at least once a day. If this be not done, the pessary, no matter of what material it may be composed, is liable to become more or less encrusted with salts deposited from the vaginal and uterine secretions. Such encrustations are nearly always found upon pessaries which have remained in the vagina for a considerable length of time. The pessary is rendered rough and irritating by their presence, and is hence much more liable to produce injury by friction and pressure. By the use of the daily injection such concretions are removed, and their accumulation is rendered impossible. Moreover, the hot injections obviate any tendency to engorgement of the pelvic blood vessels, and thus operate as a prophylactic against inflammation.

There are two classes of cases in which mechanical support for the womb may be properly resorted to, viz.:

1. Those in which the displacement of the womb is due to a remediable cause, and the mechanical support is a temporary expedient for relieving unpleasant symptoms during the time required for the removal of that cause.

2. Those in which the cause cannot be ascertained, or being ascertained, cannot be successfully treated.

This classification is simply a confession of the imperfection of the science of gynecology, and as that science progresses, the number of cases in the second class will gradually be reduced to a minimum.

In this classification it will be observed that no place is provided for cases in which mechanical support is supposed to act as a curative agent. This omission is intentional, for while theoretically such cases are possible, yet in practice I believe them to be extremely rare. I have yet to see the first case of uncomplicated displacement of the uterus which required only retention in its proper position to effect a cure. If a perfectly healthy uterus be taken from a dead body and held by the cervix between the thumb and finger, and pressure be applied to the fundus, either vertically or laterally, it will be found very difficult to produce a flexion of the organ. It is only when some portion of its tissues has undergone a pathological change that a flexion will occur. So again, to speak of an uncomplicated version of the womb would be to imply an effect without a cause. Nature has made ample provision for the support of the womb, and it is only when the ligaments are pathologically stretched, contracted or weakened, or the normal relations of the womb to neighboring parts are destroyed, that a version can occur. Hence, in this classification no place is left for uncomplicated displacements.

In the first class of cases the pessary cannot be relied upon as a curative measure. I long ago adopted the views recently expressed by Mundé, when he says, "Pessaries, even the latest and most efficient, that of Gehrung, do not act as curative means; they are merely retentive agents."* It is for the sake of temporary relief that a pessary is applied in those cases in which treatment can be directed toward removing the cause of the displacement. Such being the case, the desideratum in the pessary is obviously not a permanent adaptability to the condition of the vagina and uterus. As the result of treatment in such cases, the condition of the pelvic viscera is constantly undergoing a change, if progress be made toward a cure. Consequently, the pessary which is best adapted for use to-day may become entirely unfit for use to-morrow or next week. The change from day to day will be very slight, and yet, slight as it is, it must be met by a corresponding change in the pessary employed. There is constant danger of doing the patient harm by failing to make the proper adjustment, and thus in a few hours may be lost all the ground which has been gained in a month. The pessaries of hard rubber, block tin, glass, celluloid, etc., which are commonly used, do not meet the indications in these cases for the following reasons:

1. In the majority of cases which apply for treatment, there is inflammation in the pelvic cellular tissue. For reasons already explained, it is not safe to insert a pessary where inflammation exists. Yet, it is frequently necessary in such a case to support the womb on account of the pain produced by its traction upon the ligaments, or its pressure upon neighboring organs. Mechanical support is indicated, yet no pessary meets the indication.

2. Even though there may be no inflammation present, it will rarely fail to be the case that hardness in the roof of the vagina will show that inflammation has previously existed, which needs only a very slight irritation to kindle it anew. To avoid this danger, it is necessary to wait until this symptom has entirely subsided

*Minor Surgical Gynecology. New York, 1885, p. 209.

before introducing a pessary. Meanwhile the patient remains unrelieved of her most troublesome symptoms.

3. Even with the best of skill and attention, in a certain proportion of cases ulceration in the vaginal walls will result from the use of a pessary. In no case is it justifiable to expose a patient to this risk unless there is a certainty of obtaining a degree of benefit which will more than counterbalance it. It may well be questioned whether the temporary relief obtainable is a sufficient inducement for undertaking this risk.

4. It is impossible to adjust a pessary with the accuracy necessary to meet the requirements of a case in which the conditions are constantly changing in the process of cure, and in which the tissues are in so sensitive and inflammable a state that a slight failure in adaptation is liable to do serious mischief.

Is it necessary, then, to abandon the use of the pessary in cases where mechanical support is a means and not an end? Can any substitute be found which will neither excite inflammation nor aggravate it when already existing? In answering these questions in the affirmative, and thereby condemning the employment of pessaries in such cases, I am aware that I shall present views differing from those of a large portion of the profession. But I believe that my views are based upon correct principles, and will stand the test of clinical experiment. I have for years used a substitute which fulfills every indication, while it is not open to the objections which may be urged against the use of pessaries. The substitute referred to is the tampon of cotton. In this appliance is found a means of support for the uterus which, if properly used, can produce neither inflammation, ulceration nor pain. It may be placed in a vagina whose roof is as hard as a board and as tender as a boil, and no harm will follow. It may distend the vagina to its utmost limit, and yet the integrity of the mucous membrane will remain unimpaired. If inserted with an ordinary degree of discretion and judgment, it will produce no pain, but will communicate a comfortable feeling of support to the pelvic organs which very few pessaries can give. In skillful hands it will fulfill every requirement for the support of the uterus, while in the hands of the unskillful and inexperienced, it is incapable of doing harm.

To properly apply a tampon for the support of the uterus, it is necessary first to ascertain the position in the pelvis at which the womb is most free from pain. This may be done in the manner already pointed out. The patient then having been placed in Sims' position, and Sims' speculum having been introduced, a pledget of cotton saturated with glycerine is placed in the posterior *cul de sac*, another in the anterior fornix, and a third and fourth on either side. The size of these pledgets must be such as to fill the space between the cervix and the vaginal wall on all sides with a moderate degree of distention. The cervix may thus be held fixed with a tolerable degree of firmness in its normal axis. A larger pledget of cotton, also wet with glycerine, is placed immediately below the cervix so that it will lie between it and the vaginal promontory. The size of this pledget should be such that when in position it will raise the womb to the point where it is most free from pain and, resting upon the vaginal promontory, will form a platform of support for the cervix. A certain amount of experience is necessary in order to insert the cotton in such a manner that it will remain where it is placed and hold the womb in the desired position. If the cotton be packed too firmly around the cervix, the natural mobility of the womb will be abolished and the jar of every motion will be felt in the pelvis by the patient. On the other hand, if too loosely applied, it will be found in a few hours to have fallen down into the lower part of the vagina and all its usefulness will be gone. A certain amount of experience will enable the operator to avoid these two extremes.

A tampon of cotton should never be allowed to remain in the vagina for more than twenty-four hours. In the majority of cases which require its use, the hot vaginal injection is also indicated by the presence of inflammation. If the injection is used with the tampon in the vagina, it is robbed of all its beneficial effect, as it fails to come in contact with the cervix and the surrounding parts of the vagina. It is, therefore, necessary that the cotton should be removed before taking the injection. In order to carry out this plan, it is best that the injection should be given at the physician's office, or that the patient should be seen at her home. If she remove the cotton, take the injection, and then walk or ride to the physician's office with the womb unsupported, the good effects of the tampon will be lost.

The difference in the manner in which the cotton must be packed to adapt it to the different varieties of displacement is not as great as might be supposed. Yet it is only by an appreciation of these slight differences that the maximum of benefit from this mode of treatment can be obtained. The cardinal point involved is to so graduate the pressure of the cotton as to bring the cervix as nearly as possible into its normal axis, so that the counter-pressure from the portion of the tampon resting upon the vaginal promontory will be exerted in the direction of that axis. If the above-described manner in which the evil effects of displacement are produced be borne in mind, it will be readily understood how a slight degree of elevation in the pelvis will relieve those symptoms. This is the only result which can be hoped for from mechanical treatment. Whether the condition be that of flexion or of version, whether backward or forward, the elevation of the womb in the pelvis will give the temporary relief which must be recognized as the ultimatum of mechanical treatment. Having accomplished this, the proper means must be sought for the removal of the cause of the displacement.

The second class of cases, those in which the cause of displacement cannot be recognized, or being recognized cannot be cured, furnishes, therefore, the proper field for the employment of pessaries. These cases are the *opprobria medicorum* of gynecology. Their existence is a constant reminder of the imperfection of our art, and their treatment, even with the methods which experience has shown to be best adapted to such cases, is far from satisfactory in its results. Procidentia of long standing, in which all the supports of the uterus have been completely relaxed for many years, displacement due to interstitial or subperitoneal fibroids or other incurable tumors in the pelvis, are illustrations of this class. In such cases the adaptation of mechanical means for the support of the womb usually constitutes the whole treatment, since it is impossible to remove the cause. It is important, however, that before the case should be relegated to this class of incurables, every means should be employed which offers any hope of effecting a cure. The operations of elytrorraphy, episiorraphy, colporraphy and hysterectomy have been carried to so high a degree of success, and improved methods of operating have so far enhanced the benefits to be derived from them, that many cases, which a few years ago were regarded as amenable only to palliative remedies, are now treated with a degree of success which is eminently satisfactory. These radical operations are among the most brilliant achievements of modern surgery, and no physician has performed his full duty to his patient who has not given her the opportunity of enjoying their benefits. When they fail, as unfortunately they sometimes will, there still remains as a further resource the use of the pessary to palliate the evils which cannot be cured. I do not propose to discuss the use of pessaries in this class of cases, as little, if any, difference of opinion exists in regard to the propriety or the methods of their use. Unsatisfactory as are the results obtained, they are the best means available for the treatment of this class of cases, and as such should receive their due share of attention. It is probable that the further development of the science of gynecology will gradually lessen the number of such cases, and this prospective benefit should furnish an additional incentive to their thorough and persevering study.

